# The Antibiotic Andrimid Produced by *Vibrio coralliilyticus* Increases Expression of Biosynthetic Gene Clusters and Antibiotic Production in *Photobacterium galatheae*

**DOI:** 10.3389/fmicb.2020.622055

**Published:** 2020-12-22

**Authors:** Yannick Buijs, Thomas Isbrandt, Sheng-Da Zhang, Thomas Ostenfeld Larsen, Lone Gram

**Affiliations:** Department of Biotechnology and Biomedicine, Technical University of Denmark, Lyngby, Denmark

**Keywords:** biosynthetic gene cluster, *Vibrionaceae*, antibiotics, natural product discovery, stress response

## Abstract

The development and spread of multidrug resistant pathogens have reinforced the urgency to find novel natural products with antibiotic activity. In bacteria, orphan biosynthetic gene clusters (BGCs) far outnumber the BGCs for which chemistry is known, possibly because they are transcriptionally silent under laboratory conditions. A strategy to trigger the production of this biosynthetic potential is to challenge the microorganism with low concentrations of antibiotics, and by using a *Burkholderia* genetic reporter strain (Seyedsayamdost, Proc Natl Acad Sci 111:7266–7271), we found BGC unsilencing activity for the antimicrobial andrimid, produced by the marine bacterium *Vibrio coralliilyticus*. Next, we challenged another marine Vibrionaceae, *Photobacterium galatheae*, carrier of seven orphan BGCs with sub-inhibitory concentrations of andrimid. A combined approach of transcriptional and chemical measurements of andrimid-treated *P. galatheae* cultures revealed a 10-fold upregulation of an orphan BGC and, amongst others, a 1.6–2.2-fold upregulation of the gene encoding the core enzyme for biosynthesis of holomycin. Also, addition of andrimid caused an increase, based on UV-Vis peak area, of 4-fold in production of the antibiotic holomycin. Transcriptional measurements of stress response related genes in *P. galatheae* showed a co-occurrence of increased transcript levels of *rpoS* (general stress response) and andrimid induced holomycin overproduction, while in trimethoprim treated cultures attenuation of holomycin production coincided with a transcriptional increase of *recA* (SOS stress response). This study shows that using antimicrobial compounds as activators of secondary metabolism can be a useful strategy in eliciting biosynthetic gene clusters and facilitate natural product discovery. Potentially, such interactions could also have ecological relevant implications.

## Introduction

Microbial natural products have provided humankind with a broad range of medically useful molecules (Patridge et al., 2016). These natural products, or secondary metabolites, play important physiological and ecological roles for the producing microorganism and can shape microbial communities (Pishchany and Kolter, 2020). Due to the alarming development and spread of multidrug resistant pathogens (Michael et al., 2014), there is a need to discover novel bioactive products, in particular, novel natural products with antibacterial activity to supplement our array of clinical effective antibiotics (Moellering, 2011). In bacteria, genes encoding for the enzymes necessary to biosynthesize a natural product are almost exclusively clustered together on so-called biosynthetic gene clusters (BGCs) (Walsh and Fischbach, 2010). This genetic organization makes it possible to query the potential for production of secondary metabolites in genome-sequenced bacteria using specialized bioinformatics tools (Medema et al., 2011). Genome mining studies have revealed that the majority of BGCs have not yet been linked to a molecule, prompting the term “orphan BGCs” (Cimermancic et al., 2014; Machado et al., 2015b; Jensen, 2016). For instance, the natural products of 25 out of 35 BGCs in *Streptomyces galbus* strain KCCM 41354 remain to be discovered (Blin et al., 2019). Analogously, the red pigment prodigiosin is the only compound that could be identified from *Pseudoalteromonas rubra* strain S4059 culture extract although genome mining identified 16 BGCs, excluding the prodigiosin cluster (Vynne et al., 2011; Paulsen et al., 2019). Revealing the chemical products from orphan BGCs is an important point at the agenda of discovering novel natural products and potential novel drugs.

One commonly put-forward explanation for the high number of orphan BGCs is that, under the laboratory conditions used, these BGCs are transcriptionally silent (Rutledge and Challis, 2015). Examples of culture condition dependent production of secondary metabolites (Giubergia et al., 2016; Romano et al., 2018; Tomm et al., 2019) suggest indeed that the right culture variables can lead to activation of otherwise silent BGCs and expression in the native host will greatly facilitate further studies. One condition that can activate the production of secondary metabolites is the presence of exogenous small molecules (Mitova et al., 2008; Craney et al., 2012; Seyedsayamdost, 2014; Okada and Seyedsayamdost, 2017; Xu et al., 2017; Moon et al., 2019a; Lozano et al., 2020). As one of the earliest examples, 18 small molecules out of a 30,569 compound-large library increased the production of pigmented secondary metabolites in *Streptomyces coelicolor* (Craney et al., 2012). Unable to rely on a phenotypic read out such as pigment production, Seyedsayamdost used a genetic reporter system in a similar high throughput screen of a chemical library of approximately 600 compounds to activate production of the virulence factor malleilactone by *Burkholderia thailandensis* (Seyedsayamdost, 2014). Nine compounds in sub-minimal inhibitory concentrations (sub-MIC) activated the silent *mal* cluster in *B. thailandensis* and all of the nine inducing compounds are antibiotics targeting DNA or peptidoglycan synthesis. Applying the so-called HiTES (High Throughput Elicitor Screening) strategy has yielded at least 13 novel natural products, mostly from Gram-positive *Actinobacteria* (Xu et al., 2017, 2019; Moon et al., 2019a, b). These studies show the potential to use “old” antibiotics as a tool to un-silencing orphan BGCs. It also poses the question of what the ecological advantage for bacteria could be to increase secondary metabolite output in the face of chemical danger.

The competition sensing hypothesis addresses this question, and predicts that bacteria can employ their stress responses to sense competition and use this as a cue to increase their own antibiotic production (Cornforth and Foster, 2013). For example, in the prolific antibiotic producing genus *Streptomyces*, competing strains modulate the antibiotic output of a focal strain by chemical cues (Abrudan et al., 2015; Westhoff et al., 2020). Likewise, *Chromobacterium violaceum* increases production of the purple pigment and antibacterial violacein when it is challenged with sub-MICs of antibiotics targeting the polypeptide elongation step in protein synthesis (Lozano et al., 2020). This response was mediated by a newly identified two-component regulatory system. In *B. thailandensis*, sub-MICs of trimethoprim change intracellular metabolite concentrations, which leads to the overproduction of malleilactone (Li et al., 2020). These examples add to our knowledge on how, on a mechanistic and molecular level, sub-MICs of antibiotics induce physiological and transcriptional responses in microbes (Andersson and Hughes, 2014).

The marine Gram-negative *Vibrionaceae* family is a relatively underexplored source of natural products despite their widespread antagonistic behavior (Gram et al., 2010; Rypien et al., 2010; Wietz et al., 2010; Cordero et al., 2012) and genomic potential for secondary metabolite production (Machado et al., 2015b). We have demonstrated that *Vibrio coralliilyticus* strain S2052 and *Photobacterium galatheae* S2753 produce the antimicrobial compounds andrimid, inhibitor of fatty acid synthesis (Freiberg et al., 2004), and holomycin, a potential intracellular zinc chelator (Chan et al., 2017), respectively (Wietz et al., 2010). Both species belong to the *Vibrionaceae* family and have similar ecological niches in the marine environment (Takemura et al., 2014; Labella et al., 2017). Genome mining of the two bacteria has identified six and seven orphan BGCs in their genomes (Machado et al., 2014, 2015b). The purpose of the present study was to employ a sub-MIC antibiotic challenging strategy, aimed at activating BGC transcription for potential drug discovery purposes. Starting with the reporter strain used in Seyedsayamdost (2014), we report BGC inducing activity for the antibiotic andrimid from *V. coralliilyticus*. Challenging *P. galatheae* with andrimid resulted in a transcriptional increase of two of seven orphan BGCs and an increase in production of the antibacterial holomycin.

## Materials and Methods

### Bacterial Strains, Media and Culture Conditions

Five marine bacterial strains capable of producing antibacterial compounds (Geng et al., 2008; Mansson et al., 2011a; Vynne et al., 2011) were screened for their ability to induce expression of the silent BGC (see below): *V. coralliilyticus* S2052, *P. galatheae* S2753, *P. rubra* S4059 and *Pseudoalteromonas luteoviolacea* S4060 were isolated on the Galathea3 expedition (Gram et al., 2010; Wietz et al., 2010; Vynne et al., 2011; Machado et al., 2015a) and *Phaeobacter inhibens* DSM 17395 was isolated from seawater from scallop larvae (Ruiz-Ponte et al., 1998; Table 1). Furthermore, a *P. galatheae* holomycin-deficient mutant (Δ*hlmE*), was used as a control strain in transcription measurements (see below). The marine bacteria were cultured at 25°C on Marine Agar (MA, Difco 2216) and in Marine Broth (MB, Difco 2216) or Artificial sea water Peptone Yeast extract (APY, modified from Lefèvre et al. (2010), containing per liter milliQ H_2_O: 19.45 g NaCl, 5.9 g MgCl_2_.6H_2_O, 3.24 g Na_2_SO_4_, 1.8 g CaCl_2_.2H_2_O, 0.55 g KCl, 3.0 g HEPES, 5.0 g peptone and 3.0 g yeast extract, pH = 7.0). *B. thailandensis malL-lacZ* was kindly provided by M. Seyedsayamdost, Princeton University, and used as reporter strain for silent BGC eliciting activity assays (Seyedsayamdost, 2014). *B. thailandensis malL-lacZ* was cultured at 30 °C on LB-agar (Difco, 244520) and in LB-broth (Difco, 244620) supplemented with 50 mM MOPS. Unless stated otherwise, all bacterial growth in liquid media was carried out in shake flasks with a working volume of 1/5 of total shake flask volume, and aeration was achieved by an orbital shaker (diameter = 20 mm) at 200 rpm.

**TABLE 1:** Marine bacterial strains tested for silent BGC inducing activity with the *Burkholderia* reporter strain.

Species	Strain	antiSMASH hits	Antimicrobial compounds (Type)	Mechanism of action	References
*Vibrio coralliilyticus*	S2052	8	Andrimid (NRPS/PKS)	Fatty acid synthesis inhibition	Freiberg et al., 2004; Gram et al., 2010; Wietz et al., 2010
*Photobacterium galatheae*	S2753	10	Holomycin (NRPS)	Intracellular metal (zinc) chelator	Gram et al., 2010; Wietz et al., 2010; Chan et al., 2017
*Pseudoalteromonas rubra*	S4059	16	Prodigiosin (NRPS/PKS)	Membrane permeabilization	Gram et al., 2010; Vynne et al., 2011; Suryawanshi et al., 2017
*Pseudoalteromonas luteoviolaceae*	S4060	18	Violacein (indole)	Membrane permeabilization	Gram et al., 2010; Vynne et al., 2011; Cauz et al., 2019
*Phaeobacter inhibens*	DSM17395	8	Tropoditheitic acid (troponoid)	Proton motive force collapse	Brock et al., 2014; Wilson et al., 2016

### Generation of Holomycin-Deficient *P. galatheae* Mutant

The holomycin deficient mutant Δ*hlmE* was generated by a two-step crossover in-frame deletion strategy (Zhang et al., in preparation). In brief, an 1.0 kb upstream and downstream region flanking of the core holomycin biosynthetic gene *hlmE* were amplified and cloned into the *sacB* containing suicide vector pDM4 (Milton et al., 1996) by restriction ligation to form pDM4-del-*hlmE*. Recombinant plasmid was transferred into *E. coli* WM3064 by electroporation (Wang et al., 2016; Yin et al., 2017; Chen et al., 2018). Conjugational transfer of the suicide vector was achieved by bacterial mating of WM3064 cells carrying suicide plasmid and *P. galatheae* cells at a 1:1 ratio (based on OD600, OD600 = 0.4–0.6) for 4 h at 37°C. Counter selection of the donor strain was achieved by exploiting the diaminopimelic acid auxotrophy of *E. coli* WM3064 (Δ*dapA*); first crossover *P. galatheae* mutants were selected on chloramphenicol containing APY-agar plates without diaminopimelic acid. Second crossover mutants were obtained by single colony plating on APY-agar plates containing 10% (w/v) sucrose based on counter selection against the *sacB* gene, which is present on pDM4. Deletion mutants were confirmed by PCR and sequencing and abolished holomycin production of the *hlmE* deletion mutant cultures was confirmed by UHPLC-HRMS.

### Bacterial Culture Extracts and Filtered Supernatants

Marine bacteria were cultured and extracted after 24 h, except for *P*. *inhibens* DSM 17395, from which samples were collected after 48 h. Aliquots of 1 mL were centrifuged for 1 min at 10,000 × *g* and the supernatant sterile filtered. In parallel, 10 mL of culture broth was extracted with an equal amount of ethyl acetate. After evaporation of the solvent under N_2_, extracts were re-dissolved in 300 μL methanol.

### Reporter Strain Luminescence Assay

A *B. thailandensis* transposon mutant with an in-frame *lacZ* gene insertion in the *malL* coding sequence (*B. thailandensis malL-lacZ)* was used as reporter strain for silent BGC eliciting activity assays (Gallagher et al., 2013; Seyedsayamdost, 2014). Activation of the *mal* cluster was determined by measuring β-galactosidase activity with the luminescence-based Beta-glo assay (Promega) in 96 well plates (Corning, 3610). *B. thailandensis malL-lacZ* was inoculated at an OD600 of 0.05 in 100 μL LB-MOPS, to which 5 μL supernatant of *P. rubra* S4059, 25 μL supernatant (all other strains),1 μL ethyl acetate culture extracts dissolved in methanol, 15 μM trimethoprim or 8 μM purified andrimid (see below) were added. Control cultures were prepared by adding equal amount of solvent to the wells. The 96 well plate was wrapped in parafilm and incubated at a microplate shaker (Fisher Scientific, cat. no. 88861023) at 30°C for 24 h. Fifty μL of Beta-glo reagent was mixed with the well cultures by pipetting and the luminescence was measured in a plate reader (SpectraMax i3, Molecular Devices) after 1 h incubation in the dark. Luminescence was normalized by the OD600 values of cultures, measured prior to addition of the reagent.

### Andrimid Purification

Four liters of *V. coralliilyticus* culture grown in APY medium were collected after 24 h of aerated growth at 25 °C. The culture was extracted 1:1 with ethyl acetate (EtOAc) and the organic phase was separated and dried. The dried extract was separated into 11 fractions using an Isolera One (Biotage) flash chromatographic equipped with a SNAP column packed with 10 g of C18 material, using a linear gradient of milliQ water and methanol (MeOH) as mobile phases, starting at 10% and ending at 100% MeOH in 60 min at a flow rate of 15 mL/min. The fraction containing andrimid was further purified using a linear gradient of milliQ water and acetonitrile (ACN) each with 50 ppm trifluoroacetic acid (TFA) on a Dionex UltiMate 3000 HPLC system equipped with a Kinetex C18 column (Phenomenex) to give semi-pure andrimid. Multiple batches of andrimid were purified from *V. coralliilyticus* and we observed small batch-to-batch variations in the potency of andrimid against *P. galatheae* in shake flask culture, possibly caused by small differences in purity. Growth curves of *P. galatheae* in APY medium + andrimid (Supplementary Figure 1) served to select the specific batch-dependent dose in such a way that each batch-dependent dose had a similar potency (effect on growth) against *P. galatheae*. Andrimid batch 1 had a relatively low potency and was used for preliminary experiments (concentrations ranging from 5 to 15 μM), the holomycin production dynamics experiment (7.5 μM) and the antibiotic dose response experiment (5, 10, and 15 μM). Andrimid batch 2 had a stronger potency and was used for the reporter strain luminescence assay (8 μM) and the transcription measurements (3.5 μM) by RT-qPCR.

### Genome Mining of *P. galatheae* and Design of Primers for BGCs, Housekeeping and Stress Response Genes

The genome of *P. galatheae* was mined using antiSMASH V4.0 (Blin et al., 2017) and revealed 10 putative BGCs. Eight out of 10 BGCs were selected for transcription measurements by reverse transcription qPCR (RT-qPCR), and primers were designed based on the identified core genes (such as NRPS and PKS genes). As read-out for the possible induction of stress response by andrimid, the following genes were selected: *rpoS*, general stress response (Chiang and Schellhorn, 2010); *rpoE*, membrane damage stress response (Haines-Menges et al., 2014); *recA*, SOS response/DNA damage (Sanchez-Alberola et al., 2012); *prxA*, oxidative stress response. It has experimentally been shown that *prxA* is under control of OxyR1 in *V. cholerae* and transcription is activated during oxidative stress (Stern et al., 2012; Wang et al., 2017); we confirmed that in *P. galatheae* the genetic structure of *oxyR-prxA* is similar as in *Vibrio cholerae* (Stern et al., 2012; Wang et al., 2017). Multiple housekeeping genes (*16S-rRNA*, *gyrB*, *recA*, *fur* and *dnaG*) were tested for stable transcription levels for the use as normalization reference gene (see below). Primer pairs were designed using primer3 and checked for specificity. Resulting amplicon fragments were checked for absence of secondary structure formation at 60°C. Primer sequences, melting temperatures, accession numbers and reaction efficiencies are reported in Supplementary Table S1.

### Minimal Inhibitory Concentration Measurements

Minimal inhibitory concentrations of purified andrimid, kanamycin (Sigma-Aldrich K4000) and trimethoprim (Sigma-Aldrich 92131) against *P. galatheae* were measured in 96-well plate (Thermo Scientific 262162) cultures. In biological triplicates, *P. galatheae* was cultured in 100 μL APY medium containing one of the antibiotics in a concentration range of 320 to 2.5 μM, prepared by 2-fold serial dilutions. The cultures were incubated for 24 h at 25°C using a microtiter plate shaker (Fisher Scientific, cat. no. 88861023). The MIC value was determined as the lowest antibiotic concentration corresponding to the well were no growth was visible, which was confirmed by OD measurements at 600 nm using a plate reader (SpectraMax i3, Molecular Devices).

### RNA Extraction for Transcription Measurements

For the transcription measurements, exponentially growing precultures of *P. galatheae* and Δ*hlmE* were used to inoculate three 100 mL shake flasks with fresh APY medium at an OD of 0.001. Cultures were grown aerated at 25°C. At *T* = 3 h, andrimid was added to a concentration of 3.5 μM to one culture, 1 μM (final concentration) trimethoprim was added to the second culture and an equal amount of methanol (solvent) was added to the third culture as control. At *T* = 4 h (one hour after antibiotic addition) 500 μL exponential phase samples were taken; 250 μL samples were taken at *T* = 8 h and at *T* = 24 h. Samples were immediately mixed with a 2x volume of RNAprotect Bacteria Reagent (Qiagen) and RNA was extracted by using the RNeasy kit (Qiagen) according to manufacturer instructions, using enzymatic cell lysis. After DNase treatment with Turbo DNase (Invitrogen), the RNA samples were checked for DNA contamination by regular PCR, quantified with Qubit^TM^ (Q32852, Invitrogen) and RNA quality was checked on agarose gel and by spectrophotometry on a Denovix DS-11 (DeNovix).

### Reverse Transcription qPCR

One hundred ng of RNA per sample was transcribed into cDNA using Lunascript (New England Biolabs). qPCR experiments were carried out on a Mx300P (Agilent Technologies) using Luna Universal qPCR Master Mix (New England Biolabs) with a reaction volume of 20 μL in Optical Tube 8x Strip (Agilent Technologies), using 1 μL of cDNA sample as template and a primer concentration of 0.4 μM. A dilution series of gDNA (ranging from 10 ng to 100 pg) from *P. galatheae* was used as standard curve for each primer pair, see Supplementary Table S1 for reaction efficiencies. A two-step cycle protocol was employed with an annealing temperature of 60°C. Water, no template and no reverse-transcriptase controls were included as negative controls. For normalization of the gene transcription values, multiple housekeeping genes were considered and their cycle threshold values are reported in Supplementary Table S2. The 16S rRNA gene was found to give the most stable and reliable values across all tested conditions (different growth phases and antibiotic treatment). For the calculations of the final transcription values, each Ct value was converted to a transcript number using the standard curve, and subsequently divided by [number of transcripts of 16S rRNA / 100 million]. Each qPCR reaction was measured in technical duplicate and final transcript levels are the mean of triplicates.

### Metabolome Analysis and Holomycin Detection

Ten mL samples of *P. galatheae* cultures with and without andrimid treatment were collected after 24 h and extracted as described previously. Chemical analysis and quantification was performed on an Agilent 6545 HPLC-DAD-HRMS/MS, using the settings described in Isbrandt et al. (2020). Semi-quantification of holomycin was based on the UV-Vis absorption peak area at 390 ± 10 nm. To measure the dynamics of holomycin production, micro-extractions were performed by extracting 900 μL of sampled culture broth with an equal volume of ethyl acetate, and re-dissolving the dried extract in 100 μL methanol.

### Antibiotic Dose-Response Experiment

The effect of different concentrations of the antibiotics andrimid, kanamycin and trimethoprim on the production of holomycin by *P. galatheae* was determined in an antibiotic dose-response experiment. In a pre-experiment, antibiotic concentrations ranging between 1 and 20% of the determined MIC values (MICs of andrimid, kanamycin and trimethoprim were 80 160, and 40 μM, respectively) were tested in shake flask cultures for effect on growth physiology, i.e., growth rate reduction and maximum optical density. Based on this, a range of three concentrations per antibiotic was chosen with similar degrees of effect on growth: 5, 10 and 15 μM andrimid; 20, 30, and 40 μM kanamycin and 0.2, 0.5, and 1 μM trimethoprim. *P. galatheae* was inoculated in APY medium at a starting OD600 of 0.0015 in APY medium, with addition of antibiotics after 3 h. Culture extractions and holomycin detection were performed as described above and the experiment was carried out with biological triplicates.

### Statistical Analysis

Differences in means of transcription values and holomycin UV signal peak areas between treatments (e.g., andrimid vs. control) were tested for statistical significance by a two-sample Student’s *t*-test assuming equal variances, using a null-hypothesis of equal means. 95% confidence intervals of fold changes were calculated using Fiellers method. Transcription values were normalized by the 16S rRNA gene copy number and holomycin UV signals were normalized by OD measurements to account for cell density.

## Results

### Andrimid From *V. coralliilyticus* Activates the Mal Cluster in the Genetic Reporter Strain *B. thailandensis*

**FIGURE 1:**
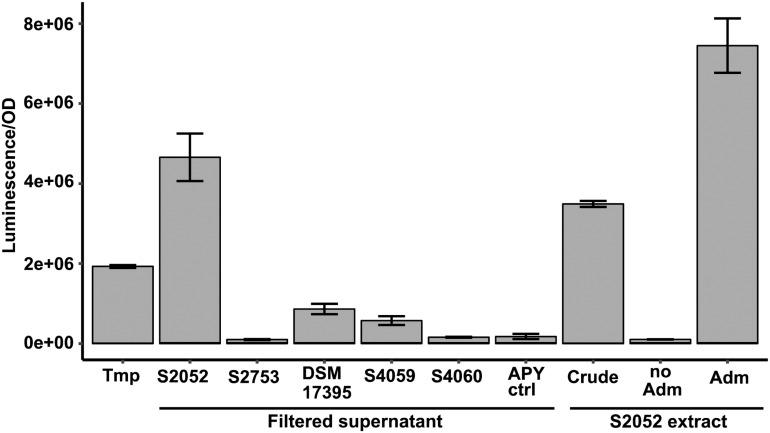
Andrimid from *V. coralliilytiucs* S2052 activates the *mal* cluster in *B. thailandensis malL-lacZ*. Luminescence signal corresponds to LacZ activity as result of *mal* cluster activation in the reporter strain. Values are normalized to OD600 and are the means of three biological replicates, error bars indicate the standard error of mean. S2052 = *V. coralliilyticus*, S2753 = *P. galatheae*, DSM 17395 = *Phaeobacter inhibens*, S4059 = *Pseudoalteromonas rubra*, S4060 = *Pseudoalteromonas luteoviolacea*, Tmp = 15 μM trimethoprim [positive control, potent elicitor in Seyedsayamdost (2014)], Adm = 8 μM andrimid. “no Adm” is the combination of S2052 extract fractions but leaving the andrimid containing fraction out.

The genetic reporter strain *B. thailandensis malL-lacZ* contains a *lacZ* fusion within the *mal* BGC that is silent under standard conditions and can therefore be used to screen conditions that activate the BGC without the need for laborious gene transcription or chemical measurements. Five antibiotic producing marine bacterial strains were tested for their capability to activate the silent *mal* BGC in *B. thailandensis*. The bacteria produce structurally different compounds (indole, non-ribosomal peptides, polyketides) with distinct antimicrobial mechanisms of actions (Table 1). Both filtered supernatant and organic extracts of the bacterial cultures were tested with the reporter strain *B. thailandensis malL-lacZ*. *V. coralliilyticus* produced a potent inducing compound of the reporter system (Figure 1) causing a 26-fold upregulation of the expression (as compared to medium control) whereas the four other strains showed weak to no induction of the *Burkholderia* reporter system. *V. coralliilyticus* produces the antibacterial compound andrimid, and we suspected that this compound was responsible for the eliciting activity. Therefore, andrimid was fractionated and purified from *V. coralliilyticus* cultures to test in sub-MICs (MIC of andrimid for *B. thailandensis malL-lacZ* was 32 μM) in the reporter strain. A combination of all extract fractions without the andrimid containing fraction did not activate the reporter system, whereas purified andrimid at a concentration of 8 μM did (Figure 1).

### BGC Transcription and Holomycin Production in *P. galatheae* Are Increased by Andrimid

To apply the sub-MIC antibiotic challenging strategy, *P. galatheae* was grown with purified andrimid. Genome mining of *P. galatheae* revealed seven orphan BGCs as well as the BGC encoding for the biosynthesis of holomycin, which is readily detectable in cultures of this strain (Table 2).

**TABLE 2:** AntiSMASH-predicted BGCs in *P. galatheae*. Bold rows indicate BGCs selected for transcriptional measurement.

BGC ID	Type	Most similar know BGC (% gene similarity)^*a*^
BGC 1	**NRPS**	–
BGC 2*^*b*^*	**NRPS**	O&K antigen (18%)
BGC 3	Ectoine	Ectoine (66%)
BGC 4	Siderophore	Aerobactin (100%)
BGC 5	**NRPS**	–
BGC 6	**NRPS/T1PKS**	–
BGC 7	**Bacteriocin**	–
BGC 8	**NRPS**	Indigoidine (80%)
BGC 9	**NRPS/T1PKS**	–
Hlm*^*c*^*	**NRPS**	Thiomarinol (18%)

*Bold rows indicate BGCs selected for transcriptional measurement. The genomic coordinates and accession numbers of the core genes of the BGCs are tabulated in Supplementary Table 1. ^a^The similarity percentage is the percentage of genes in the most similar known BGC that share significant amino acid sequence homology (BLAST e-value <1E-05; 30% minimal sequence identity; shortest BLAST alignment covers over >25% of the sequence) with genes identified in the BGC. Known BGCs are from the curated Minimum Information about a Biosynthetic Gene cluster (MIBiG) database (Blin et al., 2019). ^b^Bioinformatic analyses predict this to be the BGC responsible for Solonamide production. ^c^Experimentally confirmed to be the holomycin (Hlm) BGC.*

The effect of andrimid on the secondary metabolome of *P. galatheae* was studied by a combined approach of transcriptional and chemical measurements. First, the MIC of andrimid against *P. galatheae* was determined to 80 μM. *P. galatheae* was cultured with and without andrimid (approximately 3.5 μM, see section “Materials and Methods”), and gene transcription was measured in the exponential (*T* = 4 h; OD = 0.6–0.9), transition (*T* = 8 h; OD = 2.5–4.0) and stationary phase (*T* = 24 h; OD = 2.0–4.0) by RT-qPCR. Because holomycin is detectable in cultures of *P. galatheae*, comparing the transcription values of orphan BGCs with the transcription values of the holomycin BGC could provide clues about whether the orphan BGCs are transcriptionally silent or not.

**FIGURE 2:**
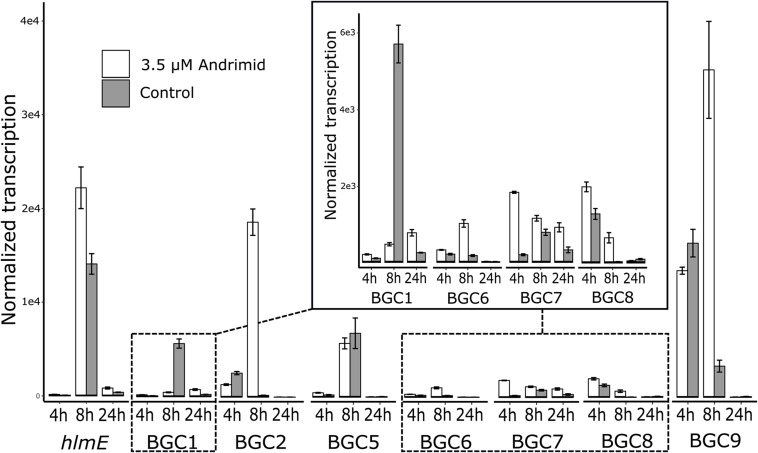
Andrimid induces transcription of BGCs in *P. galatheae*. Transcription levels of the core genes of the seven orphan and holomycin BGCs in *P. galatheae* in the exponential, transition and stationary growth phase when cultured with and without andrimid. Values are means of biological triplicates and error bars represent standard error of the mean.

**FIGURE 3:**
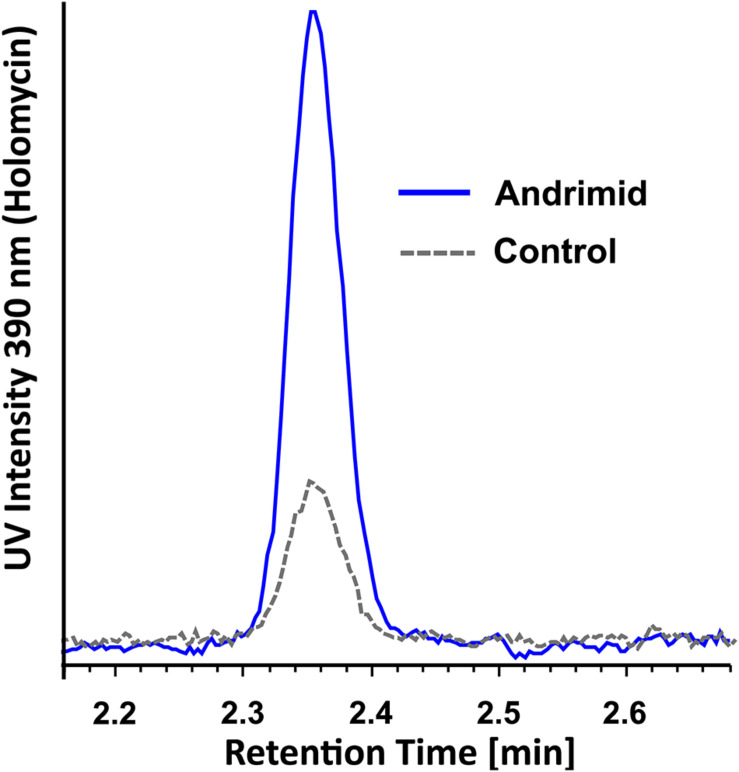
Representative overlaid UV spectra of control and andrimid treated *P. galatheae* culture extracts at 390 nm demonstrating a 4.0-fold (95% CI: 2.1–6.0, *n* = 3) increase in holomycin production.

The RT-qPCR measurements demonstrated that three orphan BGCs of *P. galatheae* had relatively low transcription values under all conditions and time points, as their maximum values were approximately 10-fold lower than the transcription value of the *hlmE* gene in the control culture at the *T* = 8 h time point (Figure 2 and Supplementary Table 3). Although we were able to detect mRNA, the expression may be insufficient to detect the biosynthetic product with the methods employed in this study. Under standard conditions (control), only BGC 9 showed a higher transcription value than the biosynthetic gene for holomycin production *hlmE* in the transition phase (*T* = 8 h), which was found to be the growth phase with the highest transcription value for this gene. Moreover, andrimid had a stimulating effect on the transcription of several BGCs, most prominently visible for BGC 2 (*p* < 0.001) and BGC 9 (*p* = 0.002), which were upregulated 102 and 10-fold respectively. Transcription levels of *hlmE* in the transition and stationary phase were moderately increased by andrimid by 1.6 and 2.2-fold (*p* = 0.031 and *p* = 0.014), which was consistent with preliminary experiments where slightly higher inductions were observed (transition phase: *p* = 0.008, stationary phase: *p* = 0.045) of 2.8-fold for both growth phases (Supplementary Figure 2). Chemical analysis of ethyl acetate extracts by HPLC-DAD and MS analysis corroborated the increased transcription levels, as we measured a 4.0 (95% CI: 2.1–6.0) fold increase in holomycin UV (390 nm) peak area for the andrimid treated cultures (Figure 3). *P. galatheae* also produces a series of cyclodepsipeptides, called solonamides (Mansson et al., 2011b; Nielsen et al., 2014). As noted earlier, the putative solonamide NRPS containing BGC (BGC 2) had a 102-fold higher transcription in the andrimid treated *P. galatheae* culture in the transition phase, but this increase did not translate to higher levels of solonamide A or solonamide B. To rule out experimental error as the cause of the discrepancy between the chemical and transcription data for the solonamides, the experiment was done a second time. Although less pronounced, this analysis showed a 9.2-fold higher transcription of the putative solonamide NRPS in andrimid treated samples, whereas the MS peak areas of solonamide A and solonamide B were similar between andrimid treatment and control cultures.

### Andrimid Increases Production of Unknown Compounds

The metabolome of *P. galatheae* cultures treated with andrimid was compared to control cultures in order to identify any differentially produced compounds (other than holomycin and solonamide A and B). Based on HPLC-DAD-HRMS analysis, minor changes were found in the metabolite profiles of the andrimid treated cultures. Specifically, six compounds (m/z values listed in Supplementary Table 4) were produced in higher amounts compared to the controls (Supplementary Figure S3). Based on database searches, using Reaxys, Antibase, and our own in-house MS/MS library, an attempt was made to match either of these to the upregulated orphan BGC 9. This BGC consists of one NRPS and Type I PKS module (Supplementary Figure S4) which predicts a rather small and simple compound consisting of one amino acid (no specific substrate was predicted for the adenylation domain) and one polyketide unit. None of the increased and unknown compounds had hits that match these criteria in any of the databases which makes it unlikely that either of these is the product of this orphan BGC.

### Holomycin Production Mainly Occurs in Transition From Exponential to Stationary Phase

**FIGURE 4:**
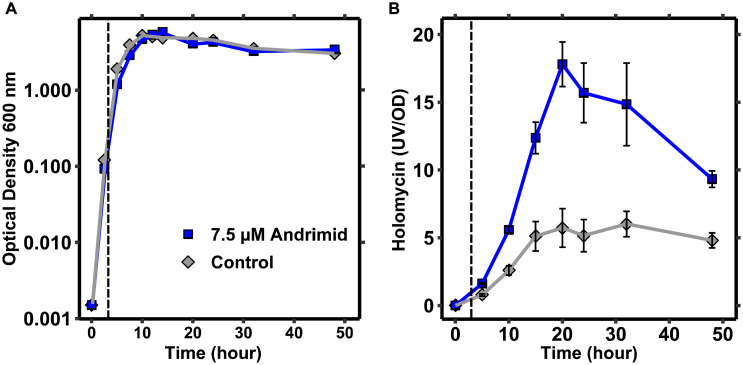
Holomycin production by *P. galatheae* occurs mainly in the transition phase from exponential growth to stationary phase. **(A)** Growth curves of andrimid treated and control *P. galatheae* cultures showing a small and temporal effect of andrimid (addition at 3 h) on exponential growth. **(B)** UV signals of micro-extractions from andrimid treated and control *P. galatheae* cultures normalized by optical density values. The dashed line marks the addition of andrimid or solvent (control) to the cultures. Values are means of three biological replicates, error bars represent standard error of the mean.

The dynamics of holomycin production by *P. galatheae* were measured to explore how andrimid induces holomycin production. Micro-extractions of culture samples combined with semi-quantification of holomycin by HPLC-DAD revealed that holomycin production was most significant in the transition from exponential to stationary growth, i.e., between 5 and 15 h given the inoculum level and growth conditions (Figure 4). Addition of andrimid to the cultures increased the production of holomycin (as observed earlier), but did not alter the production timing dynamics. *P. galatheae* may produce holomycin as protective agent against the andrimid-producing bacterium and growth of a *P. galatheae* holomycin negative mutant was compared to the growth of the wild type in the presence of andrimid, but no differences in the growth kinetics could be observed (Supplementary Figure 5).

### Antibiotic Dose Response Effect on Holomycin Production

One possible mechanism of andrimid-induced holomycin production in *P. galatheae* could be via the stress response (Cornforth and Foster, 2013). The concentration of andrimid used in the previous experiments had only a minor effect (see below) on the growth of *P. galatheae* in liquid culture: right after addition, the growth rate of the andrimid treated culture temporarily slowed down by approximately 10% as compared to the control culture, however, normal growth was resumed 4 h after andrimid addition and the culture reached a similar maximum OD (Figure 4 and Supplementary Figure 1). To test whether a general stress response could be the cause of the increased holomycin production, a dose response experiment was carried out with three different antibiotics, including andrimid. The two other antibiotics were selected based on having different modes of actions: kanamycin, targeting protein synthesis, and trimethoprim, blocking nucleic acid synthesis. Furthermore, trimethoprim was the most potent elicitor of the silent *mal* cluster in *B. thailandensis* (Seyedsayamdost, 2014).

**FIGURE 5:**
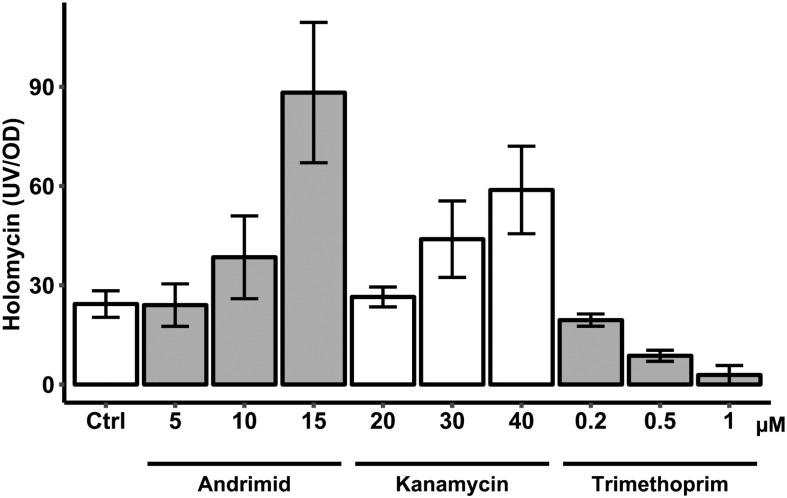
Antibiotic (andrimid, kanamycin and trimethoprim) dose response effect on production of holomycin by *P. galatheae* during a 24 h cultivation. Concentrations across the different antibiotics used had similar effects on growth of *P. galatheae*. Values are means of biological triplicates and error bars represent standard error of the mean.

Both andrimid and kanamycin increased holomycin production at higher antibiotic concentrations, whereas trimethoprim completely attenuated production of holomycin (Figure 5). Like andrimid, kanamycin increased holomycin production as compared to the control, although this was not statistically significant for the highest concentration (40 μM) used in this experiment (*p* = 0.116). Although the variation in these measurements is high, the data show that not each type of antibiotic-induced stress results in an increased holomycin production response.

### The General Stress Response May Be Involved in Holomycin Overproduction

**FIGURE 6:**
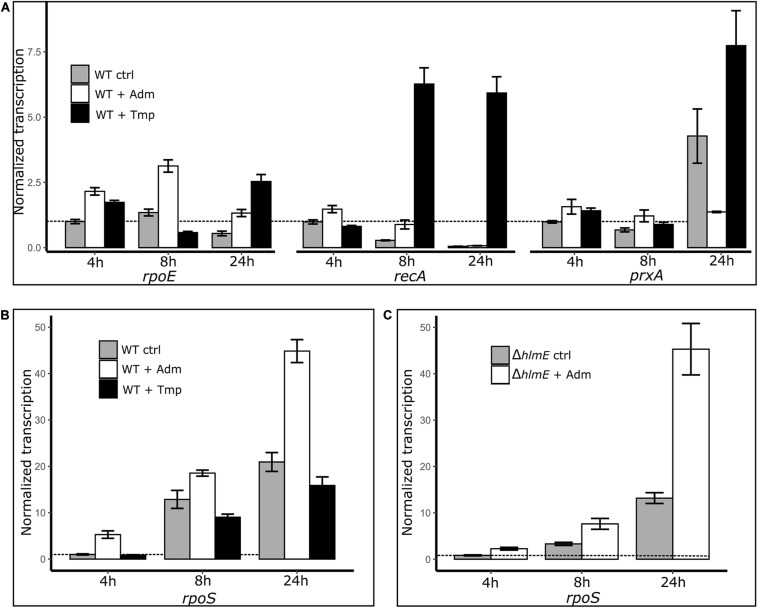
Transcriptional measurements by RT-qPCR of the genes related to stress responses: *rpoE*, membrane damage stress response; *recA*, SOS response/DNA damage; *prxA*, oxidative stress response **(A)** and *rpoS*, general stress response **(B)** in *P. galatheae* treated with andrimid (high holomycin production), trimethoprim (no holomycin production) and solvent (control), and *rpoS* in *P. galatheae ΔhlmE* a holomycin deficient mutant, treated with andrimid and solvent **(C)**. Addition of treatments was at 3 h after inoculation. For visualization purposes, the transcription values are normalized to the control transcription value at 4 h (dashed line) for each gene separately (hence: gene transcription values of different genes are not comparable in this graph, see Supplementary Tables 5, 6 for transcription values without gene-specific normalization). Values are means of biological triplicates and error bars represent the standard error of the mean.

To further investigate a possible link between the stress response and the andrimid-induced overproduction of holomycin, we measured the gene transcription levels of *rpoS*, *rpoE*, *recA* and *prxA* as read-outs of the general, membrane damage, SOS and oxidative stress responses, respectively, by RT-qPCR. Besides a control culture (only solvent addition), a trimethoprim treated culture of *P. galatheae* served as additional control, as trimethoprim induced stress did result in an attenuation of holomycin production (Figure 5). Both andrimid (3.5 μM) and trimethoprim (1 μM) had a small growth-inhibiting effect (Supplementary Figure S6). Most notably, transcript levels of *rpoS* were consistently increased in the andrimid treated cultures but not in the trimethoprim treated cultures, which showed highly increased transcription levels of *recA* (Figures 6A,B). Compared to the control, andrimid induced *rpoS* transcription significantly in the exponential (5.3-fold, *p* < 0.001) and stationary phase (2.1-fold, *p* = 0.002), but only a minor (non-significant) 1.3-fold increase was measured in the transition phase. These data show that trimethoprim predominantly activates the SOS response in *P. galatheae* and that andrimid-induced overproduction of holomycin co-occurs with an increased general stress response.

An increased general stress response in andrimid treated *P. galatheae* cultures could be caused not only by the exogenously added andrimid, but also by the andrimid-induced increased levels of holomycin production by *P. galatheae*. The experiment was therefore conducted with a holomycin deficient mutant strain of *P. galatheae*, Δ*hlmE*. Transcription levels of *rpoS* were similarly, induced by andrimid in the Δ*hlmE* strain (Figure 6C), showing that the induced levels of holomycin in the wild type are not contributing to the increase in general stress response.

## Discussion

High-throughput screening of “talented” microorganisms using small molecule elicitors has been developed as an effective tool for activating silent BGCs (Seyedsayamdost, 2014; Okada and Seyedsayamdost, 2017) that has provided several novel compounds (Xu et al., 2017, 2019; Moon et al., 2019a, b). In this study, we initially sought to identify BGC-awakening compounds produced by marine bacterial strains by using a reporter strain, with the subsequent aim to test if this eliciting activity would induce the transcription of orphan BGCs in bacteria isolated from similar environments. This strategy led to the finding that the acetyl-CoA carboxylase inhibitor andrimid produced by *V. coralliilyticus* has, in sub-MIC, stimulatory effects on the reporter strain and on the production of antibiotic holomycin by *P. galatheae*. Also, andrimid did indeed induce expression of at least two orphan BGCs in *P. galatheae*.

Sub-MICs of antibiotics induce several physiological and transcriptional responses in exposed microorganisms (Davies et al., 2006; Fajardo and Martínez, 2008; Andersson and Hughes, 2014). With this study, we add to the growing number of examples that sub-MICs of antibiotics can increase the output of natural product biosynthesis in bacteria. Andrimid had BGC inducing activity in both *P. galatheae* and the more distant soil betaproteobacterium *B. thailandensis*. Trimethoprim, on the other hand, did not increase holomycin production but was a potent elicitor of the *mal* cluster in *B. thailandensis* (Seyedsayamdost, 2014). A recent study reported the induction of violacein production in *Chromobacter violaceum* by antibiotics specifically inhibiting polypeptide elongation (Lozano et al., 2020). In a similar specific fashion, the compounds that increased actinorhodin production in *Streptomyces coelicolor* have in common that they target fatty acid synthesis (Craney et al., 2012). This is particularly interesting in light of the results presented here, as andrimid also targets fatty acid synthesis. Our data indicate that stimulation of holomycin production by *P. galatheae* is not limited to andrimid, but expands to the mistranslation-provoking antibiotic kanamycin as well. Opposed to pigment production, which enables high-throughput screens (Craney et al., 2012; Lozano et al., 2020), holomycin production is more difficult to quantify and a larger and broader screening of different classes of antibiotics was not performed. However, all taken together, there is a difference in specificity and types of antibiotic classes that induce production of (antibacterial) natural products.

In most of the aforementioned examples, the prime target has been drug discovery and ecological relevance has not been considered. *P. galatheae* and *V. coralliilyticus* are both members of the *Vibrionaceae* family, and both species have previously been isolated from eukaryotic marine organisms. The two strains used in this study were not co-isolated but metagenomic data show that they have overlapping environmental habitats. For example, *Photobacterium* spp. and *Vibrio* spp., including *V. coralliilyticus*, are both abundant in the microbiomes of the oyster *Ostrea edulis*, the mussel *Mytillus galloprovincialis* and in and around a shellfish hatchery (Gradoville et al., 2018; Dittmann et al., 2019; Li et al., 2019). This observation shows that it may be interesting to explore the ecological significance of antibiotic induced secondary metabolite production.

One possible mechanism of the andrimid-induced increase in holomycin production by *P. galatheae* could be via the stress response. The importance of the general stress response (via RpoS) in antibiotic production has been demonstrated for e.g., *Pseudomonas fluorescens* (Sarniguet et al., 1995). Involvement of the stress response would be in line with the concept of competition sensing (Cornforth and Foster, 2013), which suggests that bacteria are able to detect competitors by their hazardous natural products and respond with an advantageous counterstrategy, such as an increased output of their own antibiotic(s) or biofilm formation (Lories et al., 2020). Competition sensing also predicts that antibiotic production is most advantageous in the transition from exponential growth to stationary phase, which is indeed what the data from this study show (Figure 4). The possibility of the involvement of stress response in andrimid-induced overproduction of holomycin was explored by gene transcriptional measurements. This demonstrated that the general (*rpoS*) stress response is induced on the transcriptional level in andrimid treated cultures (increased holomycin production) as compared to both control and trimethoprim treated cultures (decreased holomycin production). These data could therefore indicate that *P. galatheae* uses its stress response to increase production of holomycin, but more work is needed to address the mechanism. It was recently shown that the trimethoprim-induced production of malleilactone in *B. thailandensis* occurs via the accumulation of metabolic intermediates rather than via stress response (Li et al., 2020). However, malleilactone is a virulence factor necessary for infection of the nematode *Caenorhabditis elegans* by *B. thailandensis* (Biggins et al., 2012), and therefore competition sensing via stress response may not be an ecological cue for its production.

The effect of andrimid on the BGC-transcriptome was broader than upregulation of the holomycin NRPS gene; and core genes of other BGCs showed higher transcription values as well. Most notably, one BGC (BGC2) was highly induced in the transition from exponential to stationary phase. This BGC is, based on bioinformatics predictions, likely responsible for the production of the solonamide compounds (Mansson et al., 2011b), however, experimental evidence is absent. Despite the large increase in transcription, an increase in MS-signals for solonamides A and B, which were already highly abundant in the control cultures, was not observed. Assuming BGC 2 is indeed responsible for solonamide biosynthesis, the discrepancy between detected compound and transcriptional activity of the putative biosynthetic gene may have been caused by translational and post-translational regulation processes.

An analysis of the upregulated BGC 9 showed that this cluster contains an NRPS gene consisting of an adenylation domain and peptidyl carrier protein, but no condensation domain, and a PKS gene that includes a ketoreductase domain (Supplementary Figure S4). Adenylation domain analysis software integrated in antiSMASH such as NRPSpredictor2 (Röttig et al., 2011) was unable to predict the substrate.

The results from this study add to previous reports of transcriptionally active orphan BGCs (Amos et al., 2017; Bukelskis et al., 2019; Dehm et al., 2019). We also demonstrate the importance of carefully choosing sample time points for measuring transcriptional activity; measuring only in the exponential phase and stationary phase, as done routinely (Amos et al., 2017; Giubergia et al., 2017), would have led to an incomplete picture and missed the high BGC-transcription levels of the transition phase (Figure 2). Our work additionally identifies which BGCs in *P. galatheae* have low transcriptional activity and may therefore be candidates for subsequent unsilencing attempts, for example by expression in a heterologous host or promoter swapping (Choi et al., 2018; Zhang et al., 2019).

In summary, the antibacterial compound andrimid from *V. coralliilyticus* induces transcription of biosynthetic gene clusters and increases production of antibiotic holomycin in *P. galatheae*. Further experiments demonstrated a possible role for the stress response in the induction mechanism. Activation of BGCs by microbially produced antibiotics could have an ecological relevance and be a useful tool in the search for novel drugs.

## Data Availability Statement

The original contributions presented in the study are included in the article/Supplementary Materials, further inquiries can be directed to the corresponding author/s.

## Author Contributions

YB and LG designed the study. YB performed the experiments. TI purified andrimid and performed chemical analyses. S-DZ constructed the holomycin deficient mutant. YB and LG analyzed the data and wrote the manuscript. All authors contributed to critical review and editing of the manuscript.

## Conflict of Interest

The authors declare that the research was conducted in the absence of any commercial or financial relationships that could be construed as a potential conflict of interest.
