# Supplementary material for: The Antibiotic Andrimid Produced by Vibrio coralliilyticus Increases Expression of Biosynthetic Gene Clusters and Antibiotic Production in Photobacterium galatheae
Source: Front Microbiol. 2020 Dec 22;11:622055. doi: 10.3389/fmicb.2020.622055 (PMC7793655; doi:10.3389/fmicb.2020.622055)
Supplement: Supplementary file 1 [file Data_Sheet_1.PDF]

Supplementary Material for

The antibiotic andrimid produced by *Vibrio coralliilyticus* increases expression of biosynthetic gene clusters and antibiotic production in *Photobacterium galatheae*

Yannick Buijs, Thomas Isbrandt, Sheng-Da Zhang, Thomas Ostenfeld Larsen and Lone Gram

Table S1. Primer sequences used for reverse transcription qPCR analysis in this study.

Primer name	Accesion number genebank + gene locus tag	Sequence (5' -> 3')	Melting temperature (°C)	Reaction efficiency (%)
**Fw_Holomycin**	JMIB01000043.1 EA58_20490	GGCCAATCAGACGCAGGTTG	64	95.6
**Rv_Holomycin**		ACCAAATGTCAGACGCGTGC	63.9	
**Fw_BGC1**	JMIB01000009.1 EA58_06710	GCTAGGACGGTATTGGATGGGT	63.8	105.3
**Rv_BGC1**		TGCTGATTTTGATACACGATGGCT	63.4	
**Fw_BGC2**	JMIB01000009.1 EA58_06305	CTCGGTACAATGTCGGGGCT	64.1	94.4
**Rv_BGC2**		CGTCCAGATTCGCCACCAAC	63.7	
**Fw_NRPS BGC5**	JMIB01000021.1 EA58_10630	TGCCGACCAAATTGTTTCTGCA	64	103.1
**Rv_NRPS BGC5**		AATTCGCCGCCACTCAAACC	64	
**Fw_PKS BGC6**	JMIB01000018.1 EA58_09610	GATATTGCTTCCATTGCCGCG	62.8	102.7
**Rv_PKS BGC6**		TCCGTGATGTCCAGCTGAATCA	63.9	
**Fw_core BGC7**	JMIB01000013.1 EA5807845	TGATCACCTGAGCTGGAGTTCC	63.9	97.3
**Rv_core BGC7**		CCAGATTCAGGGCTTCTTCGG	62.9	
**Fw_core BGC8**	JMIB01000003.1 EA58_01460	CCCAGACACATTACGATTTAGAGC A	63.4	99.3
**Rv_core BGC8**		TGCGAACACCGAGAACAGGA	64.2	
**Fw_PKS BGC9**	JMIB01000028.1 EA58_15590	GCACTTGAATCTGCTGGCGT	63.7	99.5
**Rv_PKS BGC9**		TGCGTGAGTGATCCAGGGTG	64.3	
**Fw_rpoS**	JMIB01000006.1 EA58_04340	CTCGTGAGCTGGCCCAAAAG	63.9	95.6

**Rv_rpoS**		CGCAGCATCCGGTTCACATC	63.6	
**Fw_rpoE**	JMIB01000006.1 EA58_04465	AGCCTCCCGGATGATTTGAAG	62.3	97.4
**Rv_rpoE**		GACAGCCCATGACTTCAGCA	62.5	
**Fw_recA**	JMIB01000006.1 EA58_04325	TGAGCCCGGCGAATCTTTCT	64.1	97.4
**Rv_recA**		CATCGTCCTGCTGTGCTTCA	62.8	
**Fw_prxA**	JMIB01000030.1 EA58_15970	TGGGCAAAGACGCTACCAGT	63.9	103.1
**Rv_prxA**		TCGCTGCCACCGATATGACG	64.8	
**Fw_16S**	JMIB01000046.1 EA58_21245	AGGGCTACACACGTGCTACA	63.3	95.8
**Rv_16S**		ACGACGTACTTTGTGGGATTCG	63	

Table S2. Cycle threshold (Ct) values of the tested reference genes and ΔCt values for exponential and stationary phase *P.galatheae* samples without treatment, and Ct values of the 16S reference genes under all experimental conditions. Each qPCR reaction was performed in technical duplicates, reported values are means of biological triplicates.

Reference gene	Exponential	Stationary	ΔCt
recA	23.20	27.15	3.96
gyrB	21.33	26.32	4.99
fur	21.05	23.91	2.87
dnaG	23.83	28.15	4.32
16S	14.76	14.91	0.15

16S	Control	Andrimid	Trimethoprim
Exponential	14.76	14.96	14.66
Transition	14.73	15.19	14.76
Stationary	14.91	15.16	15.06
Maximum ΔCt	0.54		

Table S3. Transcription values of the biosynthetic genes of *P. galatheae* cultured with 3.5 µM andrimid and methanol solvent as control. Values are the means of three biological replicates.

**BGC**	**Growth phase**	**mRNA copies / 100 milion copies 16-S rRNA ± standard error of mean**					
		**Control**			**3.5 µM andrimid**		
Holomycin	Exponential	77.3	±	7.9	156	±	10
	Transition	14100	±	1100	22200	±	2220
	Stationary	385	±	23	837	±	110
BGC #1 (NRPS)	Exponential	107	±	10	209	±	15
	Transition	5680	±	490	473	±	43
	Stationary	254	±	8.3	771	±	83
BGC #2 (NRPS, Solonamides)	Exponential	2570	±	170	1340	±	85
	Transition	183	±	27	18700	±	1400
	Stationary	10.2	±	0.57	13.7	±	1.1
BGC #5 (NRPS)	Exponential	248	±	35	475	±	41
	Transition	6830	±	1600	5740	±	580
	Stationary	68.5	±	6.6	39.3	±	7.5
BGC #6 (NRPS/PKS)	Exponential	215	±	21	326	±	10
	Transition	178	±	23	1010	±	97
	Stationary	20.5	±	3.7	26.3	±	1.0
BGC #7 (Bacteriocin)	Exponential	198	±	24	1820	±	26
	Transition	783	±	80	1150	±	74
	Stationary	325	±	74	912	±	120
BGC #8 (NRPS)	Exponential	1280	±	140	1980	±	130
	Transition	35.2	±	3.4	657	±	130
	Stationary	100.4	±	17	56.1	±	8.1
BGC #9 (NRPS/PKS)	Exponential	16500	±	1500	13500	±	380
	Transition	3330	±	630	34900	±	5200
	Stationary	87.4	±	21	36.8	±	3.7

Table S4. Mass to charge (m/z) values of the MS peaks that were increased in andrimid treated cultures of *P. galatheae*. Corresponding chromatogram traces are displayed in Fig. S3.

**m/z value $(M+H)^+$**	**Fold increase MS peak area**
343.1301	8.3
361.1791	4.3
621.3317	17.3
663.1947	4.6
859.4844	9.0
861.5007	6.1

Table S5. Transcription values of the stress response genes *rpoS* (general stress), *rpoE* (membrane damage), *recA* (SOS/DNA damage) and *prxA* (oxidative) of *P. galatheae* wild type cultured with 3.5 µM andrimid, 1.0 µM trimethoprim and methanol solvent as control. Values are the means of three biological replicates.

***P. galatheae* wild type**		**mRNA copies / 100 milion copies 16S rRNA ± standard error of mean**								
**Gene**	**Growth phase**	**Control**			**3.5 µM andrimid**			**1.0 µM trimethoprim**		
	Exponential	1500	±	180	7910	±	1200	1260	±	89
rpoS	Transition	22900	±	5500	28900	±	650	13500	±	980
	Stationary	31400	±	3000	67200	±	3700	23800	±	2800
	Exponential	19200	±	1600	41500	±	2700	33400	±	1400
rpoE	Transition	25900	±	2500	60200	±	4500	11100	±	850
	Stationary	10500	±	1700	25500	±	2600	48700	±	5100
	Exponential	6730	±	540	10100	±	930	5560	±	250
recA	Transition	1910	±	110	6050	±	1200	42700	±	4200
	Stationary	347	±	50	508	±	37	40400	±	4200
	Exponential	449	±	21	715	±	130	645	±	46
prxA	Transition	310	±	34	554	±	100	406	±	35
	Stationary	1950	±	470	623	±	11	3520	±	610

Table S6. Transcription values of the stress response genes *rpoS* (general stress), *rpoE* (membrane damage), *recA* (SOS/DNA damage) and *prxA* (oxidative) of *P. galatheae* ∆*hlmE* cultured with 3.5 µM andrimid, 1.0 µM trimethoprim and methanol solvent as control.

P. galatheae* ∆*hlmE		**mRNA copies / 100 milion copies 16S rRNA ± standard error of mean**					
**Gene**	**Growth phase**	**Control**			**3.5 µM andrimid**		
rpoS	Exponential	1260	±	150	3410	±	440
	Transition	4970	±	500	11400	±	1800
	Stationary	19700	±	1700	67800	±	8300
rpoE	Exponential	14200	±	780	22400	±	1600
	Transition	13300	±	680	39600	±	4900
	Stationary	13300	±	740	22700	±	5600
recA	Exponential	4660	±	450	5200	±	63
	Transition	1140	±	42	2210	±	230
	Stationary	275	±	31	361	±	27
prxA	Exponential	265	±	28	364	±	41
	Transition	189	±	2.1	183	±	26
	Stationary	597	±	170	223	±	49

Batch 1, 7.5 µM

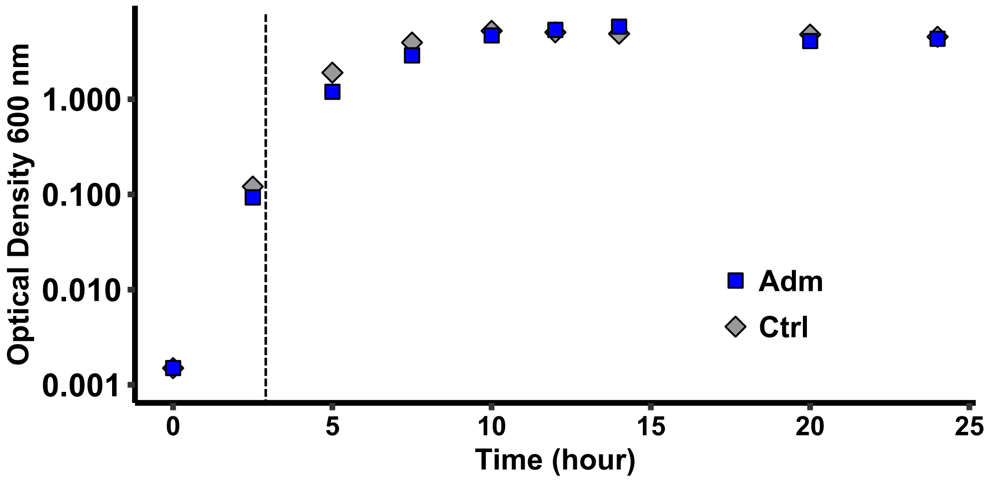

Batch 2, 3.5 µM

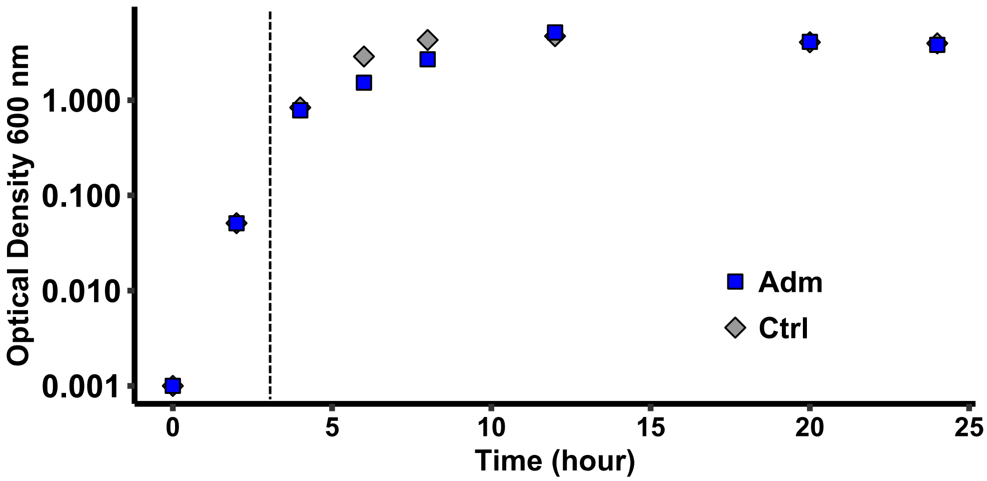

Figure S1. Growth curves of *Photobacterium galatheae* cultured with and without andrimid-batch specific doses showing an approximate similar (temporal) growth-inhibiting effect. Andrimid batch 1 was used for preliminary experiments at various concentrations, the holomycin production dynamics experiment (7.5 µM, Fig. 4) and the antibiotic dose response experiment (5, 10 and 15 µM, Fig. 5). Andrimid batch 2 was used for the reporter strain luminescence assay (8 µM, Fig. 1) and the transcriptional measurements by RT-qPCR (3.5 µM, Fig. 2 and Fig. 6). Andrimid was added after 3 hours (dashed vertical line) and data points represent the mean of three biological replicates.

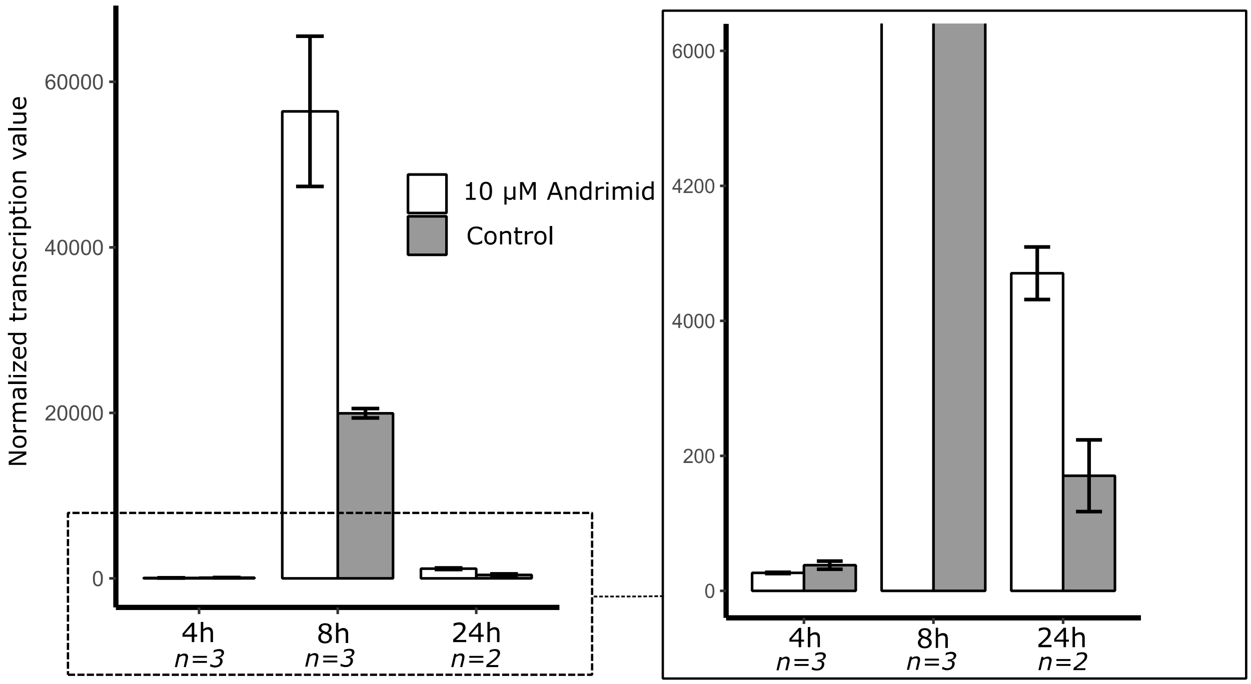

Figure S2. Data from a preliminary experiment measuring the effect of andrimid on the transcriptional activity of *hlmE*. Andrimid causes a 2.8 fold induction in the transition and stationary phase. This shows that, although the transcriptional increases for *hlmE* in Fig. 2 are not large, the andrimid inducing effect is reproducible.

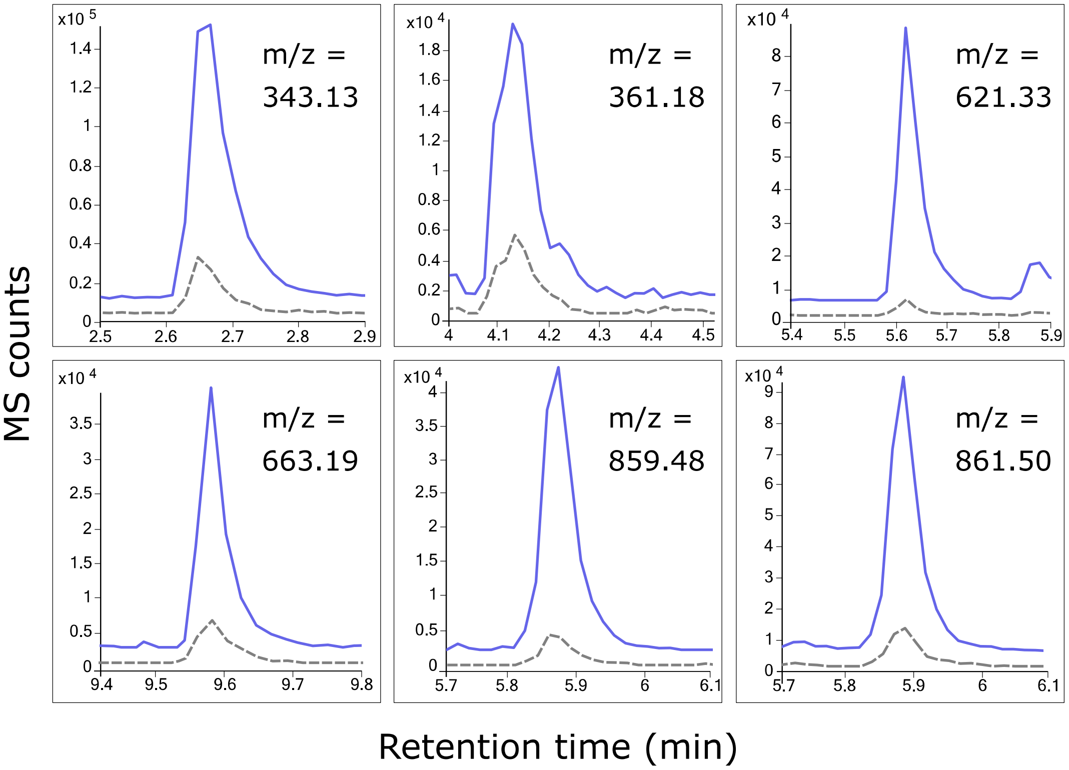

Figure S3. Representative overlaid Extracted Ion Chromatograms (EICs) of control (grey, dashed trace) and andrimid (blue) treated *P. galatheae* culture extracts at various m/z values demonstrating the induced peak areas listed in Table S4.

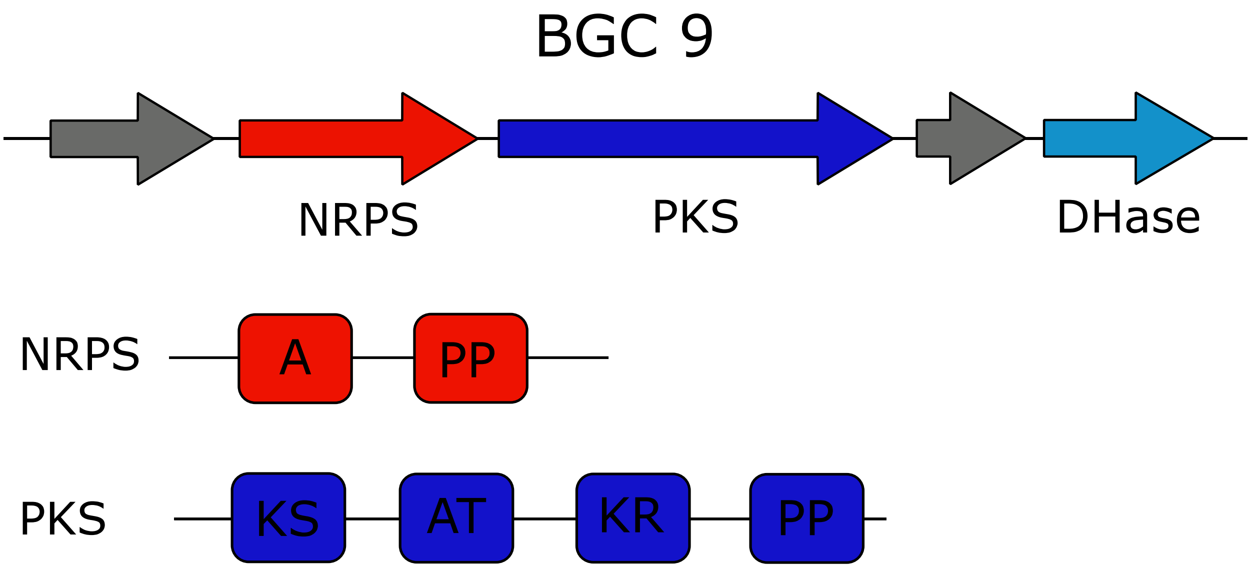

Figure S4. Architecture of BGC 9 and the biosynthetic domains of the NRPS and PKS genes. NRPS: Non Ribosomal Peptide Synthase, PKS: Polyketide Synthase, DHase: Dehydrogenase, A: Adenylation, PP: Phosphopantetheine acyl carrier Protein, KS: Ketosynthase, AT: Acyl Transferase, KR: Ketoreductase

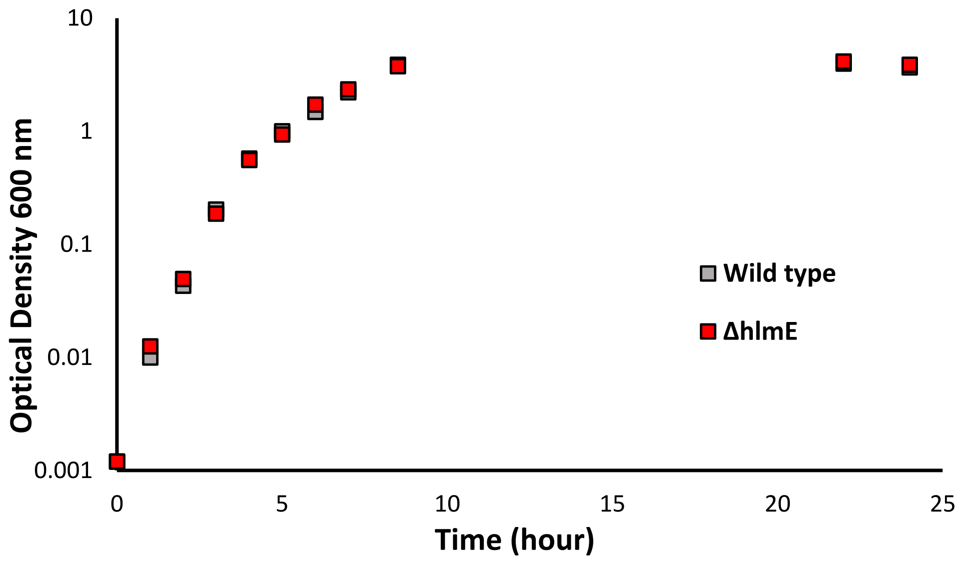

Figure S5. Growth curves of *Photobacterium galatheae* wild type and the holomycin deficient mutant (Δ*hlmE*) when cultured in APY medium + 10 µM andrimid. The ability of *Photobacterium galatheae* to produce holomycin does not influence its growth physiology when challenged with andrimid. Datapoints represent the means of two biological replicates.

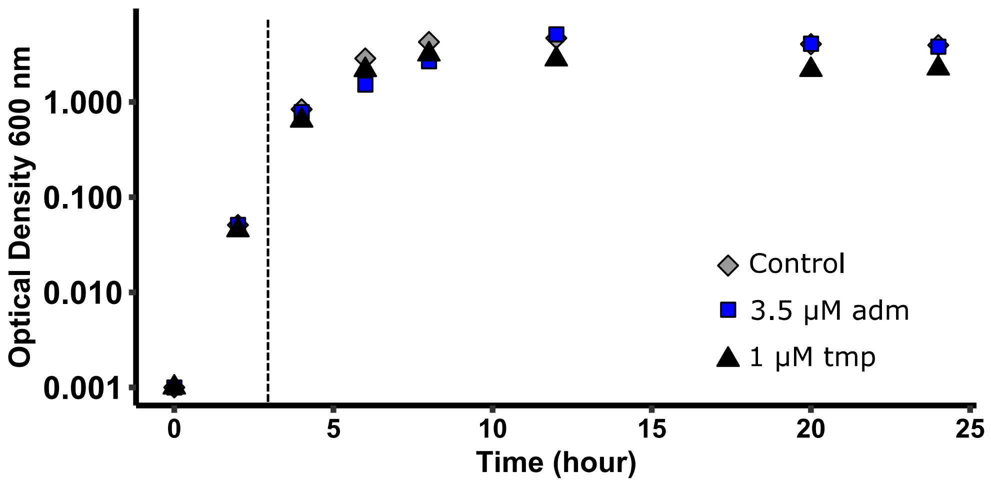

Figure S6. Growth curves of the *P. galatheae* cultures treated with 3.5 μM andrimid and 1 μM trimethoprim, corresponding to the stress response transcriptional measurement experiment (Fig. 6). Dashed line indicates the time point of antibiotic additions (T = 3h) and data points are means of three biological replicates.
